# Challenges in examining area effects across the life course on physical capability in mid-life: Findings from the 1946 British Birth Cohort

**DOI:** 10.1016/j.healthplace.2011.11.007

**Published:** 2012-03

**Authors:** Emily T. Murray, Humphrey Southall, Paula Aucott, Kate Tilling, Diana Kuh, Rebecca Hardy, Yoav Ben-Shlomo

**Affiliations:** a MRC Unit for Lifelong Health and Ageing, University College and Royal Free Medical School, 33 Bedford Place, London WC1B 5JU, United Kingdom; b Laboratory of Epidemiology, Demography, and Biometry, Gateway Building, Bethesda, MD, USA; c Great Britain Historical GIS Project, Department of Geography, University of Portsmouth, United Kingdom; d School of Social and Community Medicine, University of Bristol, Bristol, United Kingdom

**Keywords:** Life course, Area, Capability, Methodology, Deprivation

## Abstract

A major limitation of past work linking area socioeconomic conditions to health in mid-life has been the reliance on single point in time measurement of area. Using the MRC National Survey of Health and Development, this study for the first time linked place of residence at three major life periods of childhood (1950), young adulthood (1972), and mid-life (1999) to area-socioeconomic data from the nearest census years. Using objective measures of physical capability as the outcome, the purpose of this study was to highlight four methodological challenges of attrition bias, secular changes in socio-economic measures, historical data availability, and changing reporting units over time. In general, standing balance and chair rise time showed clear cross-sectional associations with residing in areas with high deprivation. However, it was the process of overcoming the methodological challenges, which led to the conclusion that in this example percent low social class occupations was the most appropriate measure to use when extending cross-sectional analysis of standing balance and chair rise to life course investigation.

## 1 Background

There is a large and growing body of literature showing that persons who live in socioeconomically ‘deprived’ areas have worse health than those who live in better areas, even after adjustment for their own socioeconomic position (Diez-Roux and Mair, 2010). But most studies focus on specific illnesses, such as cardio-vascular disease, not overall physical ability. A few studies have documented that living in a more deprived area in adulthood is associated with higher self-rated functional limitations (Basta et al., 2007), mobility disability (Lang et al., 2008), and general disability (Beard et al., 2009, Freedman et al., 2008) in late adulthood. Only one study of which we are aware (Lang et al., 2008) has investigated area effects on an objective measure of physical capability, walking speed, which is only one of a series of measures that can be used to test the ability of an individual to undertake the tasks of daily living.

Most existing studies of area effects on health rely on measures of areas assessed at a single point in time in adulthood (Diez-Roux and Mair, 2010). If over time persons move to better or worse areas or the areas in which they live change, using a single measure of residence could underestimate the effects of areas on physical capability. There is mounting evidence that physical capability in later life is a reflection of factors occurring across the life course, including growth and motor coordination in childhood (Kuh et al., 2006). In addition, numerous studies have shown that higher area deprivation is related to less availability of healthy foods and physical activity resources (Lackshman et al., 2010, Larson et al., 2009, Lovasi et al., 2009, Sallis and Glanz, 2009), higher rates of smoking (Lackshman et al., 2010, Matheson et al., 2011), and alcohol consumption (Lackshman et al., 2010, Matheson et al., 2011; Pollack et al., 2005), all of which are major risk factors for lower body functional limitations (LaCroix et al., 1993). Hence, the socioeconomic environment in which individuals live at multiple points in the life course may be associated with physical capability in later life.

Investigating the impact of area socioeconomic conditions over the life course on health in later life is complex, requiring residential addresses for each study member at multiple dates over a long period, plus corresponding area level data for each date. Any such analysis must address a number of methodological challenges.

First, in longitudinal studies drop-out inevitably occurs due to death, emigration, and refusal to participate. Some further cases are lost because old addresses cannot be located or linked to exposure data. Consequently, it is important to compare the area socioeconomic characteristics of those included and those excluded from analysis to examine if attrition bias may have occurred.

Second, the UK has experienced extensive social change over the last fifty years, including large increases in standards of living (Hicks and Allen, 1999). If we are to disentangle effects of areas at different stages of the life course, there must be enough variation in deprivation measures across areas in all years, and there need to be enough individuals moving between areas with different characteristics.

Third, consistent area level information at different points of the life course, including childhood, is required. Assessment of area deprivation in cross-sectional studies is either by direct assessment of areas by survey teams (Raudenbush and Sampson, 1999; Diez-Roux and Mair, 2010), surveys of residents (Mujahid et al., 2007; Mujahid et al., 2008; Auchincloss et al., 2009) or aggregate data, notably from the census (Riva et al., 2007; Diez-Roux and Mair, 2010). Surveys are impractical in longitudinal studies, so we must rely on census data and other official statistics. Area deprivation is often defined using indices such as the Index of Multiple Deprivation (IMD) (ODPM, 2004), Townsend deprivation index (Townsend et al., 1988), or the Carstairs deprivation index (Carstairs, 1995), which are comprised of items from the census. However, only 5 of the 7 census variables used in the Townsend or Carstairs indices were collected for census years earlier than 1971. It is therefore necessary to investigate whether single area deprivation measures, which are available at all time points, or new indices based on measures available at all time points, are associated with physical capability in the same way.

Fourth, the area measures need to be available for equivalent geographical units over time. The smallest geographical unit for which area socioeconomic data are available before 1971 is the local government district, although their average size was substantially smaller than modern districts and unitary authorities. Smaller ward units as well as local government district areas are available for more recent years. An understanding of the extent, if any, of the underestimation of area effects on physical capability when using the larger geographic boundary of local government district is required.

This research forms part of the Healthy Ageing across the Life Course (HALCyon) collaborative research programme funded by the New Dynamics of Ageing programme (www.halcyon.ac.uk). This component aims to understand how healthy ageing, as measured by physical and cognitive capability, is affected by where people live across whole of their lives. This paper uses data from the Medical Research Council (MRC) National Survey of Health and Development (NSHD), a birth cohort of men and women born in 1946, with prospectively collected residential addresses available across the whole of the life course. We link addresses collected at ages 4, 26, and 53 years to census area socioeconomic data in 1951, 1971, and 2001, respectively, to investigate the impact of four methodological challenges on moving from cross-sectional to longitudinal analyses of area effects on health in later life.

## 2 Methods

### 2.1 Study participants

The MRC NSHD is a socioeconomically stratified sample of 5362 singleton births during one week in March 1946. Cohort members have been followed up to 23 times since birth and a wealth of medical and socioeconomic data has been collected throughout the life course (Wadsworth et al., 1992). At 53 years, 3035 men and women still alive and residing in England, Scotland, and Wales were interviewed in their own homes by a team of trained nurses. The sample remains representative, in most respects, of the British-born population of the same age (Wadsworth et al., 1992, Wadsworth et al., 2003).

### 2.2 Linkage of historical census data to the National Survey of Health and Development

**Fig. 1:**
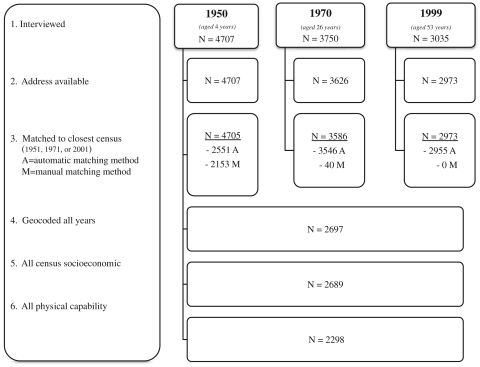
Flow of cohort member's addresses included in the analyses, by study year.

At every data collection, the address of the current place of residence was recorded for each survey member. Three ages were chosen to represent area in childhood (1950—aged 4 years), early adulthood (1972—aged 26 years), and midlife (1999—aged 53 years). Of the 2973 cohort members who had an address collected at age 53 years (1999), 2954 could be assigned a 2001 local government district (LGD) and ward (ONS, 2001a) using the UK Office for National Statistics’ (ONS) all-fields postal directory (ONS, 2001b) (Fig. 1).

For the 1972 study year, 3626 postcodes were assigned LGD units for the 1971 census (Registrar General for England and Wales (RG for EW), 1971, Registrar General (RG) for Scotland, 1971). As the postcode system was only first introduced in 1959, the full addresses from 1950 (*n*=4707) were utilized for 1951 census year LGD units (Registrar General Office, 1956a, Registrar General Office, 1956b). Initially, Imperial College London's Small Area Health Statistics Unit (SAHSU) matched residential data against the Ordnance Survey's Address-Point database (2010a) to create grid coordinates for 2551 of the 1950 addresses (54.2%) and 3586 of the 1972 postcodes (98.9%). Then, coordinates were linked to LGD units by University of Portsmouth's Great Britain Historical GIS Project based on county administrative diagrams published by the Ordnance Survey (Gregory and Southall, 1998, Gregory et al., 2002).

For the 2154 addresses from 1950 (45.8%) and 40 postcodes from 1972 (1.1%) that could not be matched at SAHSU, the GB Historical GIS team employed manual methods of LGD assignment. First, the Ordnance Survey's 1:50,000 Scale Gazetteer was used to identify addresses (Ordnance Survey, 2010b), which was successful in matching all remaining 1972 postcodes and 1432 of the 1950 addresses. For the remaining unmatched 1950 addresses, 558 were matched by Ordnance Survey County Series: 1:10,560, 1:2500, and 1:2250 sheets from the 1940s and 1950s (Registrar General for England and Wales (RG for EW), 1932–3, Registrar General for England and Wales (RG for EW), 1954) through EDINA Digimap service, then 140 modern equivalent addresses were identified in Google and matched against Ordnance Survey's Code-Point database (Ordnance Survey, 2010c). In the 23 remaining cases, previously assigned approximate 10 km grid references were used to assign an LGD. Overall for years 1950, 1972, and 1999, >99% were linked to an appropriate LGD area. This resulted in 2697 cohort members with an area assignment for all three years.

### 2.3 Life course area deprivation measures

Seven area socioeconomic measures were considered as potential proxies for area deprivation: five of which had previously been used in either the Townsend or Carstairs indexes (low social class, unemployment, overcrowding, renters, and no car) (Carstairs, 1995, Townsend et al., 1988) and two of which had been found in previous literature to be associated with functional impairment in later adulthood (lacking household amenities and higher education) (Basta et al., 2007; Freedman et al., 2008). For each defined geographic area, ‘low social class’ was defined as the percentage of all occupied persons (among males only in 1951 and 1971) in the geographic area with occupation classes 4 (partly skilled) or 5 (unskilled); ‘unemployment’ as the percentage of all economically active persons unemployed; ‘renters’ as the percentage of households that socially or privately rented; ‘no car’ as the percentage of households with no car or van; ‘lacking household amenities’ as the percentage of households without exclusive use of all amenities asked about that year (1951: hot water, stove, sink, water closet, and bath; 1971: hot water, fixed bath, and inside water closet; 2001: bath, water, and toilet); ‘overcrowding’ as the percentage of households with over 1 person per room; ‘lacking higher education’ as the percentage of all persons lacking higher level qualifications [1951: terminated education at age 20 or over (most likely age category at which degree obtained); 1971: holding a degree or equivalent; 2001: level 4 or 5 out of five (degree level qualification)] (General Register Office for Scotland, 2001, Registrar General Office, 1956a, Registrar General Office, 1956b, Registrar General for England and Wales (RG for EW), 1971, Registrar General (RG) for Scotland, 1971, Office for National Statistics, 2001a). Of cohort members with an area assignment for all three years, only 9 individuals were living in areas with no census area socioeconomic data available for at least one measure (1971: *n*=8, 2001: *n*=1).

### 2.4 Measures of physical capability

During home visits at age of 53 years (1999), participants performed three tests of physical capability: chair rise, standing balance, and grip strength. Each measure has been shown to be predictive of disability and mortality in older age groups (Cooper et al., 2010; Rantanen et al., 1999; Guralnik et al., 2000), but each test represents a separate functional component of overall physical capability (Kuh et al., 2005). Chair rise was measured as the time taken in seconds to rise from a sitting to standing position and then sit down again ten times. Standing balance as the longest time, up to a maximum of 30 s, that the cohort member could maintain a one-legged stand with eyes closed. Grip strength was measured in kilograms (kg) using an electronic handgrip dynamometer. The highest value from the maximum of four grip measures was utilized (Kuh et al., 2005). Of the 2689 persons with area socioeconomic data for all three years, 390 (14.5%) were either unable to perform (*n*=171) or missing (*n*=219) one or more of the tests. This resulted in a final sample size of 2299 cohort members with area socioeconomic data for all three years and all three physical capability tests.

### 2.5 Individual socioeconomic position

At the age of 53 years cohort member's occupations were assigned to one of the six Registrar General group classifications (Galobardes et al., 2006). Individual socioeconomic position (SEP) was based on a three category grouping of: low (classes IV and V), medium (III manual and III non-manual), and high (I and II). To make individual SEP more comparable with the categorization used in area low social class (LSC), a binary variable was also created with low SEP (manual) compared to high SEP (non-manual).

### 2.6 Analytical strategy

For challenge 1, we examined attrition bias by comparing characteristics of persons who were included and those excluded from the cross-sectional analyses in 1950 and 1972 using ANOVA for continuous variables and chi-square statistics for categorical variables. These included their LGD level area socioeconomic characteristics, country of residence (England, Scotland, or Wales), individual LSC, and gender.

For challenge 2, variability in the area level measures was investigated across time using three different approaches. First, separately for each year, the population distribution of each socioeconomic measure was assessed, with normality tested by the Shapiro–Wilk method. Second, the maintenance of relative area socioeconomic position over time was examined using quartile movement across study years by splitting individuals into groups by quartile of area deprivation at each time point and assessing their movement between these groups from 1950 to later years. Third, an exploratory factor analysis using principal axis factoring was utilized to determine whether all area measures represented an underlying common factor. The Kaiser criterion of >1 was used to determine the number of factors at each study year, then a factor matrix was used to examine the factor loadings (Norman and Streiner, 2003).

Challenge 3 was to investigate the impact of a lack of availability of area census socioeconomic data before the 1971 census. Using the study year 1999 only, a series of cross-sectional linear regressions were fitted relating area socioeconomic measures and the physical capability outcomes. Analyses were carried out for each physical capability measure as a continuous outcome. Grip strength was adjusted for body size by dividing strength in kilograms by height in centimeters, and then multiplied by 100 for presentation purposes. Due to skewed distributions, a log transformation was applied to standing balance and chair rise to achieve normality. Generalized estimating equations were used to account for clustering of individuals within areas.

Separately for each area measure, the magnitudes of effects were compared for those available at all three study years (low social class, unemployment, lacking higher education, overcrowding, and lacking household amenities) with those excluded because of lack of historical data (no car and renting). For the historically available area measures, new deprivation indices were created by summing *z*-scores of all possible two, three, and four measure combinations. For comparison purposes, the Townsend and Carstairs indices were also calculated. The Townsend Index is created by summing *z*-scores of logged unemployment, logged overcrowding, no car, and renters (Townsend et al., 1988); the Carstairs index as a sum of *z*-scores of male unemployment, overcrowding, no car, and low social class (Carstairs, 1995). In order to directly compare across measures, all indices were log transformed to achieve normality and standardized to have a mean of zero and standard deviation of one. All models were fitted before and after adjustment for individual SEP.

For the final challenge, we investigated whether use of a larger geographic level underestimates area effects by repeating our initial local government district analyses at the smaller ward level.

## 3 Results

For our sample at the age of 53 years (1999), the median standing balance and chair rise times were 5.0 (standard deviation (SD)=2.2) and 20.3 (1.4) s and mean height adjusted grip strength 2.2 (0.8) kg/cm. Physical capability scores were not highly correlated, with Pearson statistics of 16–17% between each pair of measures. In 1999, women were 49.5% of the sample, 40.3% of participants worked in low SEP occupations, and 84.8% lived in England, 10.1% in Scotland, and 5.1% in Wales. Overall, they lived in 94.5% of the total LGDs and 16.7% of the total wards from the 2001 census, with an average of 5.9 (3.9) persons per LGD and 1.2 (0.4) persons per ward (data not shown).

### 3.1 Challenge 1: attrition bias

**Fig. 2:**
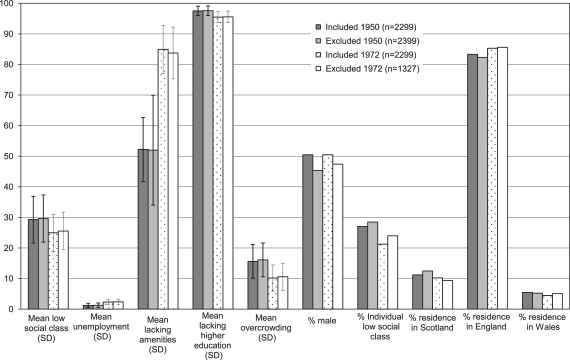
Selected area and individual characteristics of cohort members who were included and excluded in 1950 (aged 4 years) and 1972 (aged 26 years).

Of the 4705 cohort members who had a 1950 residence successfully linked to a 1951 LGD, 48.8% also had a residence in 1972 and 1999 linked to area socioeconomic data in 1971 and 2001, respectively, and performed all three physical capability tests at the age of 53 years. Only 36.6% of linked 1972 residences (*n*=1327) were excluded in the full analyses. Area socioeconomic measures from 1950 addresses were on average not different for those included and excluded (Fig. 2), while those excluded from the 1972 sample were slightly more likely to have lived in a deprived area for all area measures except higher education. Women were more likely to be excluded at both time periods, as well as those living in Scotland compared to England and Wales in 1950. Those with fathers in low SEP occupations in 1950 were less likely to be excluded but the relationship reversed for the later time period so that if a cohort member's own SEP occupation was low at age of 26 years (1972) they were more likely to be excluded than those with medium or high SEP. However, all differences were less than 5%.

Of all of the LGDs in England, Scotland, and Wales, included cohort members resided in 71.3% of districts from the 1951 census and 71.2% of districts from the 1971 census (Table 1). For both census years, districts where cohort members resided did not differ substantially from those excluded by any of the area socioeconomic measures, although they did have larger total and household populations. For the 2001 census, cohort members resided in 98.5% of LGDs with the total number of households and persons residing in LGDs increasing substantially from 1972 as a consequence of large consolidations of district boundaries.

### 3.2 Challenge 2: secular changes in area socioeconomic conditions

For the cohort members who provided residential data over 49 years of this study, between 1950 and 1999, the population average level of socio-economic conditions changed significantly. For example, when cohort members were aged 4 years (1950), the average percentage of households that were overcrowded was 47.8% (SD=17.9) with a subsequent drop to 15.1% (7.9) at age of 26 years (1972) and then 8.0% (5.1) at age of 53 years (1999) (Table 2). Similarly, the percentage of districts lacking household amenities dropped from 15.6% (5.5) at age of 4 years to 1.5% (1.1) at age of 53 years. When cohort members were aged 4 and 26 years, lacking a higher education was the norm at 97.5% (1.5) and 95.5% (1.8), respectively, with improvements to 80.5% (6.7) by the time they were aged 53 years (1999). Area low social class was the only area measure to have an approximately normal distribution at all three study periods (data not shown), although with a marked population decrease over the study years [1950: 29.4% (7.7), 1972: 25.0% (6.0), 1999: 19.8% (2.9)]. Area unemployment was low for 1950 addresses [1.2% (0.7)] and was the only measure to steadily worsen over time [1972: 2.3% (0.9); 1999: 4.7% (2.9)].

For all area socioeconomic measures, although a considerable number of cohort members retained their quartile position in the deprivation distribution across the life course, there was also a substantial amount of movement up and down the deprivation quartiles. For example, 38.1% of those residing in the highest deprivation quartile of area low social class at age of 4 years (1950) stayed in the same quartile at age of 26 years (1972) but 23.4% had moved up one quartile ranking, 22.0% two rankings, and 16.5% three rankings (Table 3). The same pattern was seen for most area measures and was true whether the cohort member began in the highest or lowest deprivation quartile. Living in an area lacking household amenities was less likely to track (31.9% stayed in highest quartile) and overcrowding was more likely to track (53.8%) from age 4 to 26 years but for both measures overall tracking declined from age 26 to 53 years.

In addition, area socioeconomic measures were not consistently correlated with each other within years and changed across study years. At age of 4 years (1950), the highest correlation was between low social class and overcrowding (spearman=0.48) (Table 4). At age of 26 years (1972), correlations between all area measures were higher than at age 4 and all measures correlated with each other (range 0.34–0.63). Most correlations between pairs of area measures at age of 53 years (1999) were lower than those at age 26 years (0.30–0.50) with no correlation between low social class and overcrowding (0.09) and slightly inverse between overcrowding and lack of higher education (−0.28). The exception was an increased correlation between low social class and unemployment (0.67).

In factor analyses only one factor was identified for each study year (Eigen value: 1950: 1.45, 1972: 2.49, 1999: 1.81) with all area measures loading onto the single factor. The strength to which an area measure loaded onto the factor varied according to measure and study year. Low social class was the only area measure to exhibit factor loadings above 0.5 for all three study years.

### 3.3 Challenge 3: availability of area socioeconomic data

In general, for each of the separate area level measures the more disadvantaged the local government district in which a cohort member resided at age of 53 years (1999), the worse their standing balance and chair rise capability at the same age, with a stronger relationship for balance than for chair rise (Table 5). For example, a 1-SD higher area low social class was associated with a standing balance time on average −10.5% (−13.7, −7.3) lower and average chair rise time 2.1% (0.7, 3.5) higher.

However, the strength of association between each area socioeconomic measure was not equal across all measures (Table 5, part I). For example, for balance the strongest associations were observed for the three area measures that had been collected across all three study periods: lacking higher education [1-SD increase: −10.3% (−13.7, −7.1)], low social class [−10.5% (−13.5, −7.1)], and unemployment [−7.5% (−10.7, −4.3)]. In contrast, relationships between overcrowding and lacking household amenities were negligible at 1.0% (−2.2, 4.2) and 1.8% (−1.4, 5.1), respectively. Associations with area measures not collected during the 1951 census, no car and renting, were between the strongest and weakest area measures at −5.6% (−8.8, −2.4) and −2.8 (−6.1, 0.4), respectively. Results were similar but weaker for chair rise time. Associations of area measures with grip strength were negligible and inconsistent.

For balance, the strongest calculated index contained the area measures of low social class and lacking higher education [−8.0% (−12.3, −3.6)] (Table 5, part II), which was not as strong as either measure modeled as a single area measure. The index containing unemployment and overcrowding was the strongest for chair rise [3.3% (1.2, 5.4)], but this also was not as strong as the singular measure of lacking higher education. The Townsend and Carstairs indices contained a mix of strong (i.e. low social class, unemployment) and weak (i.e. overcrowding) measures, resulting in estimates that were not quite as strong as the single measures of low social class and lacking higher education.

Adjustment for individual SEP at age of 53 years reduced cross-sectional associations with standing balance and chair rise times slightly but relationships remained (data not shown). Almost all associations between area measures and grip strength were attenuated to non-significance except for a strengthening of the relationship with lacking household amenities. When individual SEP was expanded to six groups, associations were hardly altered (data not shown).

### 3.4 Chapter 4: different geographical units over time

When cross-sectional associations between the 2001 district-level census area socioeconomic measures (age 53 years) and physical capability were re-run at the ward level, estimates for balance and chair rise increased (Table 6). For singular area measures, estimates for standing balance were 1.5–4.7 percentage points inversely higher and estimates for chair rise 0.2–0.9 percentage points higher. For example, a 1-SD increase of area low social class at the LGD level decreased balance time by 10.5% (7.4, 13.5) compared to the stronger association of 12.5% (9.5, 15.6) at the ward level, an increase of 2.0 percentage points or 19%. Similarly, inverse associations between higher deprivation and lower grip strength was 0.00 to 0.04 kg/cm higher at the ward than the LGD level, with the area measures of overcrowding, no car, and renting significant only at the ward geographic level [mean difference ward level (95% CI) −0.04 kg/cm (−0.07, −0.01); −0.04 (−0.07, −0.01); −0.05 (−0.08, −0.01), respectively].

## 4 Discussion

In this paper we have presented a number of challenges that need to be addressed before any investigation can be conducted on the life course area effects on health in later life. Overall, population changes in area socioeconomic conditions and changes in the data collected for the census meant that we were restricted in the area exposures which we could investigate across the lives of individuals. Yet it was in exploring these challenges that we came to the conclusion that life course area deprivation is best represented by the percentage of all occupied persons in an area with a low social class occupation; as it was the only area measure to have sufficient variability across areas, consistency of meaning, and to be consistently related to other area measures at all study years.

This is the first study to utilize geographic data of addresses collected concurrently, rather than retrospectively, during childhood, early adult, and midlife. Only one previous study using the Office for National Statistics Longitudinal Study (Curtis et al., 2004) has documented associations of poor area conditions in childhood, combining 1931 census and 1939 National Registration data, and in later adulthood, from the 1981 census, with poor health outcomes, but had no data on what happened to people between 1939 and 1981. The Atherosclerosis Risk in Communities Study (ARIC) investigated life course area effects at ages 10, 30, 40, and 50 years of age on atherosclerosis by a residential questionnaire linked to census data (Carson et al., 2007), but retrospective address collection for childhood measures potentially introduced recall bias and subsequent underestimates of effects in earlier years.

The advantage of having contemporaneously recorded addresses helped in achieving almost 100% linkage of addresses and postcodes to areas for all three study years, reducing missing data, and misclassification of area socioeconomic exposure. We were also able to document that the districts in which cohort members lived over time remained representative of districts in England, Scotland, and Wales. Similar to any longitudinal study, attrition in the MRC NSHD occurred across the life course due to death, emigration, and refusal to participate (Wadsworth et al., 2003). But unlike the two previous studies, which sampled subjects in adulthood, we were able to document that those included in the sample were not significantly different than those excluded by area socioeconomic measures during childhood and young adulthood.

The main difficulty that we encountered in preparing to investigate life course area effects on physical capability was due to changes made to the census across the different years. Two variables included in established deprivation indices – car ownership and renting – were not collected before the 1971 census. Of the measures that were collected in all years, higher education in 1951 and 1971 and lacking household amenities in 2001 had low variability across areas, making them unsuitable as proxies for area deprivation across the life course. Variations in the information each census collected were not arbitrary: the questions asked, or the way they were asked in 1951 would have made no sense in 2001. For example, in 1951 car ownership was a luxury limited to only 13% of households in Great Britain (Department of Transport, 2010) and therefore not yet of interest for a national survey. The unavailability of area socioeconomic measures in established deprivation indices meant that we had to either use a single area measure or construct our own deprivation index applied to the larger geographic level. Surprisingly, in cross-sectional analyses the creation of a composite measure did not provide any benefit over use of percent low social class in relation to any of the physical capability measures. Percentage of occupied persons in a low social class was therefore the most suitable proxy of area deprivation when examining area effects across the time period of interest in our study. This suggests that the mechanism by which ‘area’ is acting on these health outcomes may be particularly related to what class of occupation is prevalent in an area. One potential explanation is that harmful environmental factors, such as toxins, or influences on lifestyle, such as a lack of availability of fresh fruit and vegetables, are more common in certain industrial areas. It is also possible that de-industrialization has led to alterations in lifestyles and risk factors related to physical capability. For example, areas which have experienced declines of manufacturing and mining over the period of this cohort, are more likely to have higher percentages of manual occupation workers, unemployment, and lower educated populations (Ashton and Brynner, 2011) and have lower physical activity than areas that have not experienced decline (Rind et al., 2011). Further research is needed to explore the mechanisms linking the industrial composition of an area to an individual’s physical capability.

The final challenge was that area socioeconomic measures were only available at the local government district level for the 1951 census. As expected, the use of a larger geographic area did reduce the associations between almost all area measures and physical capability outcomes. This suggests that any associations observed in our life course analyses may underestimate true area effects. Changes in geographic boundaries over time limit direct comparison of area effects across years and will need to be taken into account in future life course area analyses.

In cross-sectional analyses, a clear relationship was seen between living in a socioeconomically deprived neighborhood and poorer chair rise and standing balance capability at 53 years of age. This is consistent with previous study findings that area deprivation was related to worse disability in later life (Basta et al., 2007; Bowling and Stafford, 2007; Freedman et al., 2008, Lang et al., 2008; Schootman et al., 2006). For the first time we show that associations were specific to balance and chair rise, two tests which are combinations of lower body neuromuscular speed and control (Kuh et al., 2009) as compared to grip strength, a measure of simple isometric upper body strength. This suggests that area level factors may be more important for factors that affect the central nervous functioning rather than overall muscle strength. This is consistent with previous work on the NSHD cohort that has shown that individual life course social class was related to balance and chair rise and not grip strength (Kuh et al., 2006).

Our findings, taken together with previous research, show clearly that the large individual and population changes in area socioeconomic measures mean that area effects on health need to be studied over the life course, not just cross-sectionally. For the first time we have investigated how best to deal with inconsistencies in census area socioeconomic measures over time, changes in reporting unit size to which measures apply, and secular social changes over time. Future work will incorporate these lessons to examine whether area deprivation during childhood and young adulthood contribute additional risk to poor physical capability later in life over and above the influence of individual social factors.

## Figures and Tables

**Table 1:** Mean values of local government district (LGD) characteristics for England, Scotland, and Wales: included^a^ versus excluded.

		**1950***(aged 4 years)*				**1972***(aged 26 years)*				**1999***(aged 53 years)*			
		All LGDs (*n*=1507)	Included (*n*=1074)	Excluded (*n*=433)	*p*-value	All LGDs (*n*=1406)	Included (*n*=1001)	Excluded (*n*=405)	*p*-value	All LGDs (*n*=408)	Included (*n*=402)	Excluded (*n*=6)	*p*-value
***Area socioeconomic measures***													
Low social class	30.8		30.8	30.7	0.83	25.7	25.4	26.3	0.01	19.9	19.9	20.5	0.66
Unemployment	1.1		1.1	1.0	0.01	2.2	2.2	2.1	0.65	4.7	4.8	3.6	0.16
Lacking higher education	97.4		97.5	97.1	<0.01	99.6	99.6	99.5	<0.01	80.7	80.7	75.5	0.08
Overcrowding^a^	14.1		14.7	12.7	<0.01	9.4	9.6	8.9	<0.01	1.5	1.5	1.5	0.99
Lacking household amenities	51.4		51.2	52.1	0.33	15.5	15.3	16.1	0.09	7.8	7.7	12.8	0.01
No car	–		–	–	–	–	–	–	–				
Rent	–		–	–	–	–	–	–	–				

***Area population measures***													
Number of persons		32,418	11,930	9364	<0.01	38,467	45,760	22,453	<0.01	101,320	102,327	33,844	0.01
Number of households		9288	41,713	2735	<0.01	12,968	16,697	3778	<0.01	58,462	59,047	19,276	0.01

Included are local government districts for cohort members who participated in the study year and had their address/postcode assigned to a local government district from 1951 (*n*=4698), 1971 (*n*=3618), or 2001 (*n*=2955).

a England and Wales only in 1951 and 1971 census.

**Table 2:** Distribution of area socioeconomic measures^a^ with geocoded addresses from 1950, 1972, and 1999 and physical capability outcomes at age of 53 years (*n*=2298).

	**1950***(aged 4 years)*		**1972***(aged 26 years)*		**1999***(aged 53 years)*	
	Mean (SD)	Range	Mean (SD)	Range	Mean (SD)	Range
***Area measures***						
Low social class	29.4 (7.7)	9.3–49.7	25.0 (6.0)	8.2–52.1	19.8 (2.9)	13.8–30.0
Unemployment	1.2 (0.7)	0.2–5.3	2.3 (0.9)	0.5–6.8	4.7 (1.9)	2.0–11.4
Lacking higher education	97.5 (1.5)	84.4–99.7	95.5 (1.8)	85.9–99.1	80.5 (6.7)	48.5–91.5
Overcrowding^b^	15.7 (5.5)	4.2–36.5	10.2 (4.2)	2.2–27.8	1.5 (1.1)	0.5–12.5
Lacking household amenities	48.2 (18.0)	3.4–89.5	15.1 (7.9)	1.6–50.7	7.9 (5.1)	1.6–27.3
No car	–	–	44.1 (13.0)	18.4–76.4	23.8 (9.7)	8.4–57.6
Rent	–	–	48.3 (14.8)	13.8–90.3	25.2 (8.4)	9.6–72.3

***Physical capability***						
Balance time, s^c^	–	–	–	–	5.0 (2.2)	1.0–30.0
Char rise time, s^c^	–	–	–	–	20.3 (1.4)	5.0–322.0
Grip strength, kg/cm	–	–	–	–	2.2 (0.8)	0.1–5.1

a Derived from addresses/postcodes geocoded and linked to a local government district from census years 1951, 1971, and 2001.

b England and Wales only in 1951 and 1971.

c Geometric mean (standard deviation).

**Table 3:** Of those cohort members residing in local government districts in the highest and lowest quartiles of area socioeconomic measures^a^ in 1950, their subsequent distribution (%) of quartile ranking in 1972 and 1999.

	**Highest quartile**^b^**1950***(aged 4 years)***(*****n*****=577)**		**Lowest quartile**^b^**1950***(aged 4 years)***(*****n*****=579)**	
Quartile ranking	**1972***(26 yrs)*	**1999***(53 yrs)*	**1972***(26 yrs)*	**1999***(53 yrs)*
Low social class				
0	16.5	14.2	39.7	40.2
1	22.0	21.0	29.2	28.7
2	23.4	23.9	18.5	21.9
3	38.1	40.9	12.6	9.2

Unemployment				
0	13.4	14.3	35.9	33.3
1	20.6	21.9	27.2	26.5
2	21.2	21.2	26.3	25.1
3	44.8	42.7	10.6	15.1

Lacking higher education				
0	19.9	15.5	38.3	40.4
1	15.2	19.7	29.7	26.6
2	20.9	24.6	20.4	18.1
3	44.1	40.2	11.6	14.9

Overcrowding^c^				
0	20.7	24.8	53.8	22.1
1	15.5	23.4	23.8	23.8
2	21.5	30.2	13.7	22.6
3	42.4	21.7	8.7	31.6

Lacking household amenities				
0	17.0	23.6	40.4	33.4
1	26.3	25.8	21.9	24.5
2	24.8	30.2	18.3	20.4
3	31.9	20.5	19.3	21.4

a Derived from addresses/postcodes geocoded and linked to a local government district from census years 1951, 1971, and 2001.

b Highest deprivation quartile=3; lowest quartile ranking=0.

c England and Wales only in 1951 and 1971.

**Table 4:** Area socioeconomic measures^a^ spearman correlations and factor loading, by census year (age in years).

	**Low social class**	**Unemployment**	**Lacking higher education**	**Overcrowding**^b^	**Lacking household amenities**	**Factor loading**
**1950***(aged 4 years)*						
Low social class	–	0.29	0.39	0.48	0.29	0.66
Unemployment		–	0.01	0.40	−0.02	0.39
Lacking higher education			–	0.33	0.33	0.46
Overcrowding^b^				–	0.32	0.70
Lacking household amenities					–	0.39

**1972***(aged 26 years)*						
Low social class	–	0.56	0.53	0.63	0.49	0.78
Unemployment		–	0.40	0.62	0.36	0.69
Lacking higher education			–	0.42	0.34	0.63
Overcrowding^b^				–	0.60	0.77
Lacking household amenities					–	0.63

**1999***(aged 53 years)*						
Low social class	–	0.67	0.48	0.09	0.30	0.75
Unemployment		–	0.31	0.48	0.43	0.87
Lacking higher education			–	−0.28	0.13	0.41
Overcrowding^b^				–	0.21	0.34
Lacking household amenities					–	0.45

a Derived from addresses/postcodes geocoded and linked to a local government district from census years 1951, 1971, and 2001.

b England and Wales only in 1951 and 1971.

**Table 5:** Cross-sectional associations of 2001 census area socioeconomic measures (1 standard deviation increase) with physical capability measures for cohort members' residence in 1999 *(aged 53 years)* at the local government district (LGD) geography.

	**Standing balance (s)**	**Chair rise time (s)**	**Grip strength (kg/cm)**
	Mean percentage point difference (95% CI)	Mean percentage point difference (95% CI)	Mean difference (95% CI)

***Part I***			
A. Low social class	−10.5 (−13.7, −7.3)	2.1 (0.7, 3.5)	0.03 (−0.01, 0.06)
B. Unemployment	−7.5 (−10.7, −4.3)	2.0 (0.6, 3.4)	0.00 (−0.03, 0.03)
C. Lacking higher education	−10.3 (−13.5, −7.1)	3.4 (2.0, 4.8)	0.02 (−0.01, 0.06)
D. Overcrowding^a^	1.0 (−2.2, 4.2)	−0.3 (−1.7, 1.2)	−0.04 (−0.07, 0.00)
E. Lacking household amenities	1.8 (−1.4, 5.1)	−0.6 (−2.1, 0.8)	0.05 (0.02, 0.08)
F. No car	−5.6 (−8.8, −2.4)	1.2 (−0.2, 2.6)	−0.01 (−0.04, 0.02)
G. Rent	−2.8 (−6.1, 0.4)	0.9 (−0.5, 2.4)	−0.01 (−0.04, 0.02)

***Part II***			
Townsend index	−3.7 (−6.9, −0.5)	1.1 (−0.3, 2.5)	−0.02 (−0.05, 0.01)
Carstairs index	−6.2 (−9.4, −3.0)	1.7 (0.3, 3.2)	−0.01 (−0.04, 0.02)
New potential indexes			
1. A+B	−5.3 (−9.9, −0.7)	2.3 (0.2, 4.3)	−0.02 (−0.07, 0.02)
2. A+C	−8.0 (−12.3, −3.6)	0.9 (−1.1, 2.9)	−0.04 (−0.08, 0.00)
3. A+D	−1.3 (−5.9, −3.3)	1.6 (−0.4, 3.6)	−0.04 (−0.08, 0.01)
4. B+C	−1.8 (−6.5, −2.9)	0.4 (−1.7, 2.5)	−0.01 (−0.05, 0.04)
5. B+D	−2.0 (−7.0, 2.9)	3.3 (1.2, 5.4)	−0.07 (−0.12, −0.02)
6. C+D	−1.5 (−6.1, 3.0)	1.0 (−1.0, 2.9)	−0.03 (−0.08, 0.01)
7. A+B+C	−2.1 (−6.8, −2.6)	1.5 (−0.6, 3.6)	−0.02 (−0.07, 0.02)
8. A+B+D	−2.3 (−6.8, 2.3)	0.8 (−1.2, 2.8)	−0.02 (−0.07, 0.02)
9. B+C+D	−2.4 (−7.0, −2.3)	2.3 (0.3, 4.2)	−0.03 (−0.07, 0.02)
10. ALL	−3.5 (−8.1, 1.1)	2.0 (0.0, 4.0)	−0.03 (−0.07, 0.02)

a England and Wales only in 1951 and 1971.

**Table 6:** Change^a^ in cross-sectional associations of 2001 census area socioeconomic measures (1 standard deviation increase) with physical capability measures for cohort members' residence in 1999 *(aged 53 years):* ward compared to local government district (LGD) geography.

	**Standing balance (%)**	**Chair rise time (%)**	**Grip strength (kg/cm)**
	Change in mean percentage points	Change in mean percentage points	Change in mean differences
***Area characteristics 1999***			
A. Low social class	−2.0	−0.7	0.04
B. Unemployment	−2.9	−0.5	0.02
C. Lacking higher education	−2.6	−0.2	0.02
D. Overcrowding^b^	−4.7	−0.9	0.00
E. Lacking household amenities	−1.5	−0.4	0.02
F. No car	−2.5	−0.8	0.03
G. Rent	−4.0	−0.3	0.04

a *ß*_(ward level)_–*ß*_(district level)_.

b England and Wales only in 1951 and 1971.

## References

[bib1] Ashton D., Brynner J., Wadsworth M., Brynner J. (2011). Labour market, employment, and skills. A Companion to Life Course Studies: The Social and Historical Context of the British Birth Cohort Studies.

[bib56] Auchincloss A.H., Diez Roux A.V., Mujahid M.S., Shen M., Bertoni A.G., Carnethon M. (2009). Neighborhood resources for physical activity and healthy foods and incidence of type 2 diabetes mellitus. Archives of Internal Medicine.

[bib2] Basta N.E., Matthews F.E., Chatfield M.D., Brayne C., MRC-CFAS (2007). Community-level socio-economic status and cognitive and functional impairment in the older population. European Journal of Public Health.

[bib3] Beard J.R., Blaney S., Cerda M., Frye V., Lovasi G.S., Ompad D., Rundle A., Vlahov D. (2009). Neighborhood characteristics and disability in older adults. Journal of Gerontology: Social Sciences.

[bib47] Bowling A., Stafford M. (2007). How do objective and subjective assessments of neighborhood influence social and physical functioning in older ages? Findings from a British survey of ageing. Social Science & Medicine.

[bib5] Carson A.P., Rose. K.M., Catellier D.J., Kaufman J.S., Wyatt S.B., Diez-Roux A.V., Heiss G. (2007). Cumulative socioeconomic status across the life course and subclinical atherosclerosis. Annals of Epidemiology.

[bib6] Carstairs V. (1995). Deprivation indices: their interpretation and use in relation to health. Journal of Epidemiology and Community Health.

[bib7] Cooper R., Kuh D., Hardy R., Mortality Review Group, FALCyon and HALCyon Study Teams (2010). Objectively measure physical capability levels and mortality: systematic review and meta-analysis. British Medical Journal.

[bib8] Curtis S., Southall H., Congdon P., Dodgeon B. (2004). Area effects on health variation over the life course: analyses of the longitudinal study sample in England using new data on area of residence in childhood. Social Science & Medicine.

[bib9] Department of Transport, 2010. National Travel Survey. Table NTS0205 Household Car Availability: Great Britain, 1951 to 2009. Department of Transport Statistics. Retrieved July 29th, 2011, from: 〈http://www2.dft.gov.uk/pgr/statistics/datatablespublications/nts/driving-availability/nts0205a.xls〉.

[bib10] Diez-Roux A.V., Mair C. (2010). Neighborhoods and health. Annals of the New York Academy of Sciences.

[bib11] Freedman V.A., Grafova I.B., Schoeni R.F., Rogowski J. (2008). Neighborhoods and disability in later life. Social Science & Medicine.

[bib46] Galobardes B., Shaw M., Lawlor D.A., Lynch J.W., Davey Smith G. (2006). Indicators of socioeconomic position (part 2). Journal of Epidemiology and Community Health.

[bib12] General Register Office for Scotland, 2001. Census: Standard Area Statistics (Scotland) [Census Area Statistics (CAS) Wards]. ESRC/JISC Census Programme, Census Dissemination Unit, Mimas (University of Manchester)/Centre for Interaction Data Estimation and Research (University of Leeds).

[bib13] Gregory I.N., Southall H.R., Carver S. (1998). Putting the past in its place: the Great Britain Historical GIS. Innovations in GIS 5.

[bib14] Gregory I.N., Bennett C., Gilham V.L., Southall H.R. (2002). The Great Britain Historical GIS Project: from maps to changing human geography. The Cartographic Journal.

[bib53] Guralnik J.M., Ferruci L., Pieper C.F., Leveille S.G., Markides K.S., Ostir G.V., Studenski S., Berkman L.F., Wallace R.B. (2000). Lower extremity function and subsequent disability: consistency across studies, predictive models, and value of gait speed alone compared with the short physical performance battery. Journal of Gerontology (Medical Sciences).

[bib15] Hicks, J., Allen, G., 1999. Research Paper 99/111: A Century of Change: Trends in UK Statistics Since 1900. House of Commons Library, Social and General Statistics Section.

[bib17] Kuh D., Bassey E.J., Butterworth S., Hardy R., Wadsworth M.E.J., Musculoskeletal Study Team (2005). Grip strength, postural control, and functional leg power in a representative cohort of British men and women: associations with physical activity, health status, and socioeconomic conditions. Journal of Gerontology.

[bib18] Kuh D., Hardy R., Butterworth S., Okell L., Richards M., Wadsworth M., Cooper C., Sayer A.A. (2006). Developmental origins of midlife physical performance: evidence from a British Birth Cohort. American Journal of Epidemiology.

[bib19] Kuh D., Cooper R., Hardy R., Guralnik J., Richards M., Musculoskeletal Study Team (2009). Lifetime cognitive performance is associated with midlife physical performance in a prospective national birth cohort study. Psychosomatic Medicine.

[bib20] Lackshman R., McConville A., How S., Flowers J., Wareham N., Cosford P. (2010). Association between area-level socioeconomic deprivation and a cluster of behavioural risk factors: cross-sectional, population-based study. Journal of Public Health (Oxford).

[bib55] LaCroix A.Z., Guralnik J.M., Berkman L.F., Wallace R.B., Satterfield S. (1993). Maintaining mobility in late life : II. Smoking, alcohol consumption, physical activity, and body mass index. American Journal of Epidemiology.

[bib21] Lang I.A., Llewellyn D.J., Langa K.M., Wallace R.B., Melzer D. (2008). Neighbourhood deprivation and incident mobility disability in older adults. Age and Ageing.

[bib23] Larson N.I., Story M.T., Nelson M.C. (2009). Neighborhood environments: disparities in access to healthy foods in the U.S.. American Journal of Preventive Medicine.

[bib24] Lovasi G.S., Hutson M.A., Guerra M., Neckerman K.M. (2009). Epidemiology Reviews.

[bib25] Matheson F.I., Lafreniere M.C., White H.L., Moineddin R., Dunn J.R., Glazier R.H. (2011). Preventive Medicine.

[bib48] Mujahid M.S., Diez Roux A.V., Morenoff J.D., Raghunathan T. (2007). Assessing the measurement properties of neighborhood scales: from psychometrics to ecometrics. American Journal of Epidemiology.

[bib49] Mujahid M.S., Diez Roux A.V., Shen M., Gowda D., Sanchez B., Shea S., Jacobs D.R., Jackson S.A. (2008). Relation between neighborhood environments and obesity in the Multi-Ethnic Study of Atherosclerosis. American Journal of Epidemiology.

[bib26] Norman, G.R., Streiner, D.L., 2003. Exploratory factor analysis. In: PDQ (Pretty Darned Quick) Statistics, 3rd ed. BC Decker Inc., Hamilton, London, pp. 144–155.

[bib28] Office for National Statistics, 2001a. Census: Standard Area Statistics (England and Wales) [Census Area Statistic (CAS) output areas]. ESRC/JISC Census Programme, Census Dissemination Unit, Mimas (University of Manchester)/Centre for Interaction Data Estimation and Research (University of Leeds).

[bib29] Office for National Statistics, Postcode Directories, 2001b. ESRC Census Programme, Census Dissemination Unit. Mimas (University of Manchester)/Census Geography Data Unit (UKBORDERS), EDINA (University of Edinburgh), February 2001.

[bib31] Office of the Deputy Prime Minister, The English Indices of Deprivation, 2004. ODPM Publications, London, pp. 1–180 (revised).

[bib32] Ordnance Survey, 2010a. ADDRESS-POINT: User Guide and Technical Specification. Ordnance Survey, Southampton.

[bib33] Ordnance Survey, 2010b. 1:50,000 Scale Gazetteer Technical Specification. Ordnance Survey, Southampton.

[bib34] Ordnance Survey, 2010c. Code-Point: User guide and Technical Specification. Ordnance Survey, Southampton.

[bib50] Pollack C.E., Cubbin C., Ahn D., Winkleby M. (2005). Neighbourhood deprivation and alcohol consumption: does the availability of alcohol play a role?. International Journal of Epidemiology.

[bib51] Rantanen T., Guralnik J.M., Foley D., Masaki K., Leveille S., Curb J.D., White L. (1999). Midlife hand grip strength as a predictor of old age disability. Journal of the American Medical Association.

[bib52] Raudenbush S.W., Sampson R.J. (1999). Ecometrics: toward a science of assessing ecological settings, with application to the systematic social observation of neighborhoods. Sociological Methodology.

[bib35] Registrar General Office (1956). Census 1951 England and Wales.

[bib36] Registrar General Office (1956). Census 1951 Scotland, County Reports.

[bib37] Registrar General for England and Wales (RG for EW), 1932–3. 1931 Census of England and Wales: County Reports. H.M.S.O., London.

[bib38] Registrar General for England and Wales (RG for EW), 1954. 1951 Census of England and Wales: County Reports. H.M.S.O., London.

[bib39] Registrar General for England and Wales (RG for EW), 1971. Census: Small Area Statistics (Great Britain). District information. Retrieved May 25th, 2010, from: 〈http://cdu.mimas.ac.uk/lct/〉.

[bib40] Registrar General (RG) for Scotland, 1971. Census: Small Area Statistics (Great Britain). County information. Retrieved May 25th, 2010, from: 〈http://cdu.mimas.ac.uk/lct/〉.

[bib41] Rind, E., Jones, A., Southall, H., 2011. Declining physical activity and the socio-cultural context of industrial restructuring. In: Symposium conducted at the International Medical Geographer's Conference, Durham, United Kingdom, July 2011.

[bib57] Riva M., Gauvin L., Barnett T.A. (2007). Toward the next generation of research into small area effects on health: a synthesis of multilevel investigations published since July 1998. Journal of Epidemiology and Community Health.

[bib42] Sallis J.F., Glanz K. (2009). Physical activity and food environments: solutions to the obesity epidemic. Millbank Quartlerly.

[bib54] Schootman M., Andreson E.M., Wolinsky F.D., Malmstrom T.K., Miller J.P., Miller D.K. (2006). Neighborhood conditions and risk of incident lower-body functional limitations among middle-aged African Americans. American Journal of Epidemiology.

[bib43] Townsend P., Phillimore P., Beattie A. (1988). Health and Deprivation: Inequalities and the North.

[bib44] Wadsworth M.E.J., Mann S.L., Rodgers B., Kuh D.J.L., Hilder W.S., Yusuf E.J. (1992). Loss and representativeness in a 43 year follow up of a national birth cohort. Journal of Epidemiology and Community Health.

[bib45] Wadsworth M.E.J., Butterworth S.L., Hardy R.J., Kuh D.J., Richards M., Langenberg C., Hilder W.S., Connor M. (2003). The life course prospective design: an example of benefits and problems associated with study longevity. Social Science & Medicine.

